# Comportamento Sedentário, Hábitos Alimentares e Risco Cardiometabólico em Crianças e Adolescentes Fisicamente Ativos

**DOI:** 10.36660/abc.20220357

**Published:** 2023-01-24

**Authors:** Paula Adriana dos Santos de Fontes, Jordana Herzog Siqueira, Haysla Xavier Martins, Polyana Romano Oliosa, Divanei Zaniqueli, José Geraldo Mill, Rafael de Oliveira Alvim

**Affiliations:** 1 Universidade Federal do Amazonas Faculdade de Educação Física e Fisioterapia Manaus AM Brasil Universidade Federal do Amazonas, Faculdade de Educação Física e Fisioterapia, Manaus, AM – Brasil; 2 Universidade Federal do Espírito Santo Programa de Pós-Graduação em Saúde Coletiva Vitória ES Brasil Universidade Federal do Espírito Santo, Programa de Pós-Graduação em Saúde Coletiva, Vitória, ES – Brasil; 3 Universidade Federal do Espírito Santo Programa de Pós-Graduação em Ciências Fisiológicas Vitória ES Brasil Universidade Federal do Espírito Santo, Programa de Pós-Graduação em Ciências Fisiológicas, Vitória, ES – Brasil; 4 Universidade Federal do Amazonas Departamento de Ciências Fisiológicas Manaus AM Brasil Universidade Federal do Amazonas, Departamento de Ciências Fisiológicas, Manaus, AM – Brasil

**Keywords:** Comportamento Sedentário, Fatores de Risco Cardiometabólico, Criança, Comportamento Alimentar

## Abstract

**Fundamento:**

O comportamento sedentário tem sido associado a diversos fatores de risco cardiometabólicos durante a infância. No entanto, pouco se sabe sobre o impacto do comportamento sedentário na saúde e nos hábitos alimentares de crianças e adolescentes fisicamente ativos.

**Objetivo:**

Avaliar a associação entre comportamento sedentário e fatores de risco cardiometabólicos e hábitos alimentares em crianças e adolescentes fisicamente ativos.

**Métodos:**

Esse estudo transversal avaliou 516 crianças e adolescentes (10 a 18 anos; ambos os sexos) fisicamente ativos integrados no projeto social “Estação Conhecimento-Vale”. O comportamento sedentário foi determinado de maneira indireta (questionário), utilizando como ponto de corte o tempo sentado ≥ 3 h/dia. Foram estatisticamente significantes as análises com p-valor < 0,05.

**Resultados:**

O comportamento sedentário não foi associado ao excesso de peso (odds ratio = 0,72 [intervalo de confiança (IC) de 95%: 0,325-1,389]), hipertrigliceridemia ( *odds ratio* = 0,63 [IC95%: 0,306-1,297]), HDL colesterol baixo ( *odds ratio* = 0,57 [IC95%: 0,323-1,019]) e HDL não colesterol alto ( *odds ratio* = 0,63 [IC95%: 0,283-1,389]). Contudo, crianças e adolescentes que adotavam comportamento sedentário apresentaram maior chance de consumir regularmente alimentos em frente à televisão ( *odds ratio* =1,96 [IC95%: 1,114-3,456]) e de consumir pelo menos um alimento ultraprocessado por dia ( *odds ratio* =2,42 [IC95%: 1,381-4,241]). Além disso, apresentaram menor chance de consumir frutas regularmente (OR=0,52 [IC95%: 0,278-0,967]).

**Conclusão:**

Não houve associação entre comportamento sedentário e fatores de risco cardiometabólicos em crianças e adolescentes fisicamente ativos. Contudo, o comportamento sedentário foi associado a hábitos alimentares inadequados. Dessa forma, podemos sugerir que a prática regular de atividade física pode atenuar os efeitos deletérios do comportamento sedentário nos parâmetros cardiometabólicos de crianças e adolescentes.

## Introdução

As doenças cardiovasculares são a principal causa de mortalidade no mundo e um dos principais fatores que levam à incapacidade.^1^ Estudos demonstram que a presença de obesidade e dislipidemia durante a infância e adolescência está associada ao aumento do risco de eventos cardiovasculares na vida adulta.^2^ De acordo com estudos evolvendo uma amostra representativa de adolescentes brasileiros, a prevalência de obesidade e hipercolesterolemia foi de 8,4% e 21%, respectivamente.^3 , 4^ A partir desses números alarmantes, torna-se importante a implementação de políticas públicas de saúde que visem melhorar a qualidade da alimentação, impulsionar a prática de atividade física e desestimular o comportamento sedentário durante a infância e adolescência.

O comportamento sedentário já foi considerado sinônimo de inatividade física, contudo, atualmente é definido como tempo gasto, na posição sentada, em atividades de baixo gasto energético (≤ 1,5 equivalente metabólico - MET).^5^ Atualmente, há grande preocupação com o comportamento sedentário, visto que este engloba muitas atividades nas quais crianças e adolescentes costumam realizar por longos períodos na posição sentada, tais como: uso de celular, computador, *tablet* , *videogame* e assistir à televisão. A partir disso, organizações estadunidenses de saúde ( *National Heart, Lung, and Blood Institute* e *American Academy of Pediatrics* ) têm recomendo que as crianças limitem o tempo de lazer em frente à televisão, celular e computador.^6 , 7^ Resultados de recente revisão sistemática e metanálise evidenciaram associação entre o elevado tempo em comportamento sedentário e aumento do ganho de peso, redução da aptidão física, hábitos alimentares inadequados, elevação da pressão arterial, aumento da prevalência de doenças crônicas e piora no perfil lipídico e controle glicêmico.^8 - 10^ Contudo, o impacto do comportamento sedentário no perfil cardiometabólico de crianças e adolescentes fisicamente ativos ainda é objeto de discussão.

Os hábitos alimentares, construídos e consolidados na infância e adolescência, tendem a permanecer durante toda a vida^11 , 12^ e estão associados à ocorrência de doenças crônicas na idade adulta.^13^ Nos últimos anos, tem sido observado aumento global no consumo de bebidas e alimentos ultraprocessados, bem como redução no consumo de alimentos *in natura* (frutas e hortaliças) na população infantil.^14^ As crianças que apresentam hábito de realizar as refeições em frente à tela tendem a optar por alimentos e bebidas de baixa qualidade nutricional, pois são convenientes e hiperpalatáveis.^15^ Portanto, a qualidade nutricional das refeições parece sofrer influência de comportamentos alimentares, como comer sozinho em frente à tela.^16^

Diante desse cenário e considerando que a maioria dos estudos investigam o impacto do comportamento sedentário em crianças e adolescentes fisicamente inativos, o presente estudo tem como objetivo avaliar a associação entre comportamento sedentário e fatores de risco cardiometabólicos e hábitos alimentares em crianças e adolescentes fisicamente ativos.

## Método

### Desenho do estudo e seleção da amostra

Trata-se de um estudo transversal realizado em uma amostra recrutada de um projeto social denominado “Estação Conhecimento” (Serra, Espírito Santo, Brasil), no qual compreende uma parceria entre o setor público, a mineradora VALE, e a comunidade. O projeto é direcionado a crianças e adolescentes residentes no entorno da “Estação”. A maioria das famílias que vivem nesta região pertence à classe socioeconômica mais baixa. Todos os participantes estavam regularmente matriculados em escolas públicas do município e frequentavam o projeto no contraturno da jornada escolar para terem acesso a aulas complementares e a oportunidade de participar de atividades esportivas e culturais.

**Figura 1:**
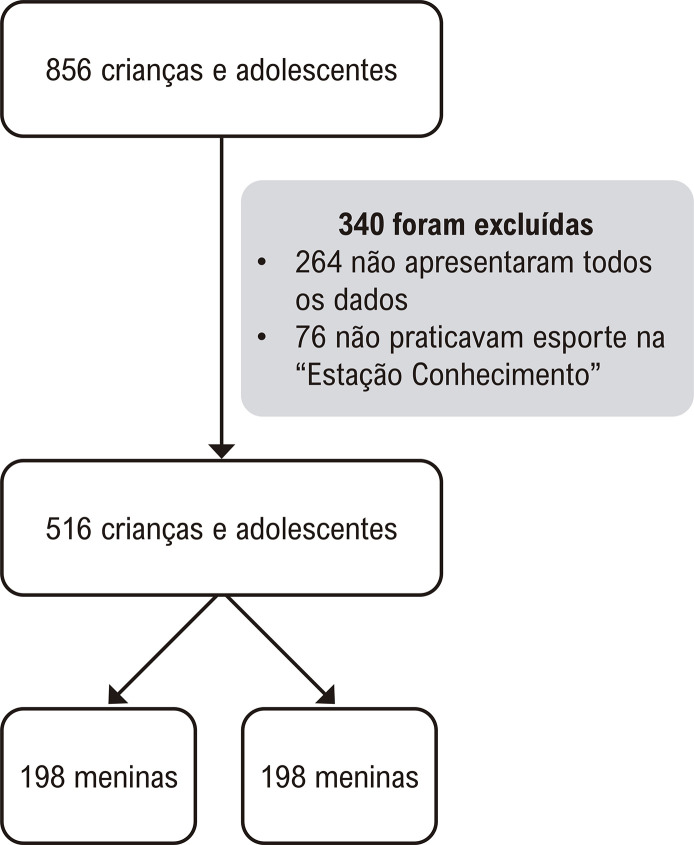
– Fluxograma da amostra.

De fevereiro de 2014 a abril de 2016, 856 crianças e adolescentes de 6 a 18 anos de idade foram atendidos na Clínica de Investigação Cardiovascular localizada na Universidade Federal do Espírito Santo, para realização de exames clínicos, bioquímicos e coleta de informações relacionadas ao estilo de vida por intermédio de questionário. Do total de participantes avaliados, 264 foram excluídos por não apresentarem algum dos dados avaliados no presente estudo (crianças com idade inferior a 10 anos não responderam os questionários relacionados à atividade física e hábitos alimentares) e 76 foram excluídos por não praticarem modalidades esportivas na “Estação Conhecimento”. Dessa forma, foram elegíveis 516 crianças e adolescentes de 10 a 18 anos de ambos os sexos ( Figura 1 ). O termo de consentimento livre e esclarecido, bem como o termo de assentimento, quando necessário, foram obtidos dos pais ou tutores antes da participação em conformidade com as normas do Comitê Institucional de Ética em Pesquisa (número 30385014.8.0000.5060).

Fonte: autores.

### Variáveis demográficas e estágio puberal

Dados demográficos e de estilo de vida foram coletados através de um questionário padrão desenvolvido pelos pesquisadores. A classificação de raça/cor foi autorreferida e avaliada por um único entrevistador. Para os pré-púberes (< 10 anos), a análise do entrevistador foi dominante em relação à raça/cor autorreferida pelo participante. Entretanto, para os púberes e os pós-púberes a informação autorreferida foi mantida no banco de dados. Pouca informação divergente foi observada em qualquer estágio puberal. Os fenótipos associados à ascendência étnico-racial como cor de pele, características do cabelo e traços faciais, foram levados em consideração para classificar o indivíduo como branco, negro ou pardo.

A Escala de Tanner foi adotada para definir as fases do desenvolvimento sexual de acordo com as características sexuais secundárias. As categorias relacionadas ao estágio puberal (pré-púbere, púbere e pós-púbere) foram elencadas considerando os critérios validados para meninos e meninas nos estudos de Marshall e Tanner.^17 , 18^

### Variáveis antropométricas

O peso foi medido em balança eletrônica com precisão de 0,05 kg (Toledo, Brasil) com os participantes descalços, usando apenas roupas íntimas e após micção. A estatura foi obtida em estadiômetro de parede (Seca Stadiometer - Seca GmBH & Co) com precisão de 0,1 cm. A composição corporal, percentual de gordura corporal e massa magra, foi avaliada por análise de impedância bioelétrica multifrequencial (MF-BIA8, InBody 230, Biospace). O índice de massa corporal (IMC) foi calculado como a razão entre o peso e a altura ao quadrado (kg/m^2^ ). Os percentis do IMC para sexo e idade foram determinados por meio das curvas padronizadas disponibilizadas pela Organização Mundial de Saúde e transformados em escore z. Os indivíduos com escore z do IMC ≥ +1 foram alocados na categoria sobrepeso/obesidade.^19^

### Variáveis bioquímicas

A coleta de sangue foi obtida em jejum de 8 a 12 horas por punção venosa no antebraço. As amostras foram processadas no próprio local da coleta e enviada para um laboratório central (Laboratório Tommasi, Vitória, Brasil) para determinar as concentrações séricas de colesterol total, HDL colesterol (HDL-c), triglicérides (TG) e glicose. O LDL-c foi calculado pela equação de Friedewald para aqueles participantes com triglicerídeos até 400 mg/dL. O não HDL-c foi calculado subtraindo-se o HDL-c do colesterol total. Foram classificados como parâmetros bioquímicos indesejáveis: TG > 90mg/dL; HDL-c < 45mg/dL; não HDL-c >120 mg/L.^7 , 20^

### Variáveis hemodinâmicas

A pressão arterial foi medida no braço esquerdo com o uso de um aparelho oscilométrico validado (Omrom 705CP, Intellisense, Japão) após um período de repouso de 5 minutos com o participante sentado na cadeira de medida. O tamanho do manguito foi escolhido após medida da circunferência do braço do participante, tomada no ponto médio entre o acrômio e o olécrano, de acordo com a recomendação do fabricante. Após o repouso, foram feitas três medidas com intervalo de 1 minuto entre elas. Quando observada variação de mais de 5 mmHg na pressão arterial sistólica (PAS) e/ou na pressão arterial diastólica (PAD), uma quarta medida foi realizada. Para obtenção dos valores da PAS e PAD, a primeira medida foi descartada, e a média entre as duas medidas mais próximas dentro da variação de 5 mmHg foi calculada. Os indivíduos com PAS ou PAD ≥ percentil 95 foram classificados como hipertensos.^21^

### Comportamento sedentário e hábitos alimentares

Os dados referentes ao comportamento sedentário e hábitos alimentares foram obtidos por meio de questões pertencentes ao questionário aplicado na Pesquisa Nacional de Saúde do Escolar.^22^ Todos os participantes do presente estudo eram ativos, pois praticavam exercícios físicos durante os treinamentos aplicados em cada modalidade esportiva (futebol, natação, judô e atletismo) dentro da “Estação Conhecimento”. Todos os participantes realizavam pelo menos 180 min/semana de exercício físico com intensidade moderada a vigorosa. A informação relacionada ao comportamento sedentário foi extraída por meio da seguinte pergunta: “Em um dia de semana comum, quanto tempo você fica sentado(a), assistindo televisão, usando computador, jogando videogame, conversando com amigos(as) ou fazendo outras atividades sentado(a)? (não contar sábado, domingo, feriados e o tempo sentado na escola)”, sendo as opções de resposta: 1) menos de 1 h/dia; 2) 1 a 2 h/dia; 3) 3 a 4 h/dia; 4) 5 a 6 h/dia; 5) 7 a 8 h/dia; 6) mais de 8 h/dia. Foram classificados como apresentando comportamento sedentário todas as crianças e adolescentes que ficavam por tempo igual ou superior a 3 h/dia^23 , 24^ realizando algumas das atividades descritas na questão acima.

Três variáveis de comportamento alimentar foram estudadas: (1) comer enquanto estudava ou assistia televisão; (2) consumo de bebidas e alimentos ultraprocessados (guloseimas, biscoitos recheados, doces, balas, chocolates, refrigerante, embutidos, salgados de pacote, salsicha, mortadela, salame, presunto, nuggets, linguiça); e (3) consumo de frutas *in natura* . O consumo diário de pelo menos 1 ultraprocessado (alimento/bebida) e comer regularmente enquanto estuda ou assiste televisão (≥ 5 dias na semana) foram considerados marcadores de comportamento alimentar inadequado. O consumo regular de frutas *in natura* (≥ 5 dias na semana) foi considerado um marcador de comportamento alimentar adequado.

### Análise estatística

As variáveis categóricas foram comparadas entre os sexos pelo teste qui-quadrado e relatadas como frequências absolutas e relativas, enquanto as variáveis contínuas com distribuição paramétrica foram comparadas pelo teste t de Student não pareado e relatadas como média ± desvio padrão. As variáveis contínuas com distribuição não paramétrica foram comparadas pelo teste de Mann-Whitney e relatadas como mediana (intervalo interquartil). A normalidade dos dados foi confirmada pelo teste de Kolmogorov-Smirnov. Análise de regressão logística binária multivariada foi utilizada para testar a associação entre comportamento sedentário (≥ 3 h/dia sentado), sobrepeso/obesidade, dislipidemia (TG e não HDL-c elevados e HDL-c baixo) e comportamento alimentar (consumo de alimentos em frente à televisão e consumo de frutas e ultraprocessados). Todas as análises foram ajustadas para sexo, raça/cor e estágio puberal. As análises foram realizadas no *software* SPSS (versão 22) (Chicago, IL, EUA), com o nível de significância fixado em 5%.

## Resultados

Foram avaliadas 516 crianças e adolescentes, sendo 318 (61,6%) do sexo masculino. As características gerais da amostra estão apresentadas na Tabela 1 . Os meninos apresentaram maior massa magra, menor percentual de gordura corporal e maiores níveis de PAS. Contudo, as meninas apresentaram maiores níveis de não HDL-c. Em relação aos hábitos alimentares, um maior percentual de meninas consome pelo menos um alimento ultraprocessado por dia.

**Tabela 1:** – Características gerais de crianças e adolescentes fisicamente ativos estratificadas por sexo

**Variáveis**	**Meninas n=198**	**Meninos n=318**	**p-valor**
**Idade**	12,7 ± 1,81	12,7 ± 1,9	0,971
**Raça/cor**			0,002*
Branco	42 (21,2)	52 (16,4)	
Preto	87 (43,9)	192 (60,4)	
Pardo	62 (31,3)	70 (22,0)	
Outros	7 (3,5)	4 (1,3)	
**Estágio puberal**			0,330
Pré-púbere	14 (7,4)	35 (11,2)	
Púbere	151 (79,5)	242 (77,6)	
Pós-púbere	25 (13,2)	35 (11,2)	
**IMC (kg/m^2^)**	19,8 ± 3,9	19,4 ± 3,6	0,261
**Gordura corporal (%)**	25,5 ± 7,7	20,2 ± 9,5	<0,001*
**Massa magra (kg)**	18,7 ± 4,6	20,6 ± 6,6	0,001*
**Sobrepeso/obesidade (%)**	42 (21,2)	55 (17,3)	0,268
**PAS (mmHg)**	103,6 ± 8,1	106,8 ± 9,2	<0,001*
**PAD (mmHg)**	62,2 ± 6,3	62,1 ± 6,8	0,900
**Hipertensão (%)**	5 (2,5)	11 (3,5)	0,552
**Glicose (mg/dL)**	85,0 ± 8,7	86,8 ± 10,6	0,066
**HDL-c (mg/dL)** ^**†**^	49 (42-55)	47 (41-54)	0,254
**HDL-c indesejável**	73 (37,4)	133 (42,6)	0,247
**Não HDL-c (mg/dL)** ^**†**^	98 (84-115)	92 (79-110)	0,008*
**Não HDL-c indesejável**	44 (22,6)	45 (14,4)	0,019*
**Triglicérides (mg/dL)** ^**†**^	67 (55-88)	63 (47-85)	0,060
**Triglicérides indesejável**	42 (21,6)	67 (21,5)	0,963
**Comportamento sedentário**			
Tempo sentado ≥ 3 horas/dia	30 (15,2)	35 (11,1)	0,168
**Comportamento e hábitos alimentares**			
Consumo regular^#^ de alimentos em frente à televisão	83 (45,4)	141 (49,6)	0,167
Consumo regular^#^ de frutas	73 (38,4)	96 (32,2)	0,187
Consumo diário de pelo menos 1 ultraprocessado	88 (47,1)	107 (35,9)	0,015*

As variáveis foram expressas em n (%) e média ± desvio padrão ou mediana (intervalo interquartil). ^*†*^ Variáveis com distribuição não paramétricas. ^*#*^ Consumo regular de alimentos e frutas foi considerado para frequência ≥ 5 dias/semana. *Diferença estatisticamente significante. HDL-c, lipoproteína de alta densidade: IMC: índice de massa corporal; PAD: pressão arterial diastólica; PAS: pressão arterial sistólica.

*Fonte: Autores.*

A Tabela 2 mostra a comparação entre os grupos quanto aos dados sociodemográficos, hemodinâmicos, bioquímicos, composição corporal e desenvolvimento puberal. O grupo que apresentou comportamento sedentário teve maior percentual de pós-púberes, maior proporção de crianças e adolescentes que consumiam pelo menos um alimento ultraprocessado por dia e que tinham hábito regular de consumir alimentos em frente à televisão. Além disso, esse grupo apresentou menor percentual de indivíduos que consumiam regularmente frutas frescas.

**Tabela 2:** – Características gerais de crianças e adolescentes fisicamente ativos estratificadas pelo comportamento sedentário

**Variáveis**	**< 3 horas n=451**	**≥ 3 horas n=65**	**p-valor**
**Sexo**			0,168
Meninas	168 (37,3)	30 (46,2)	
Meninos	283 (62,7)	35 (53,8)	
**Raça/cor**			0,150
Branco	79 (17,5)	15 (23,1)	
Preto	243 (53,8)	36 (55,4)	
Pardo	121 (26,8)	11 (16,9)	
Outros	8 (1,8)	3 (4,6)	
**Estágio puberal**			0,002*
Pré-púberes	48 (11,0)	1 (1,5)	
Púberes	344 (78,7)	49 (75,4)	
Pós-púberes	45 (10,3)	15 (23,1)	
**IMC (kg/m^2^)**	19,5 ± 3,8	19,7 ± 2,9	0,639
**Gordura corporal (%)**	22,3 ± 9,3	21,9 ± 9,4	0,703
**Massa magra (Kg)**	19,7 ± 5,2	21,3 ± 5,2	0,060
**Sobrepeso/obesidade**	87 (19,3)	10 (15,4)	0,451
**PAS (mmHg)**	105,6 ± 9,1	105,4 ± 6,9	0,901
**PAD (mmHg)**	62,3 ± 6,8	61,1 ± 4,8	0,070
**HDL-c (mg/dL)** ^**†**^	47,5 (41-55)	49 (43-54,5)	0,250
**HDL-c indesejável**	185 (41,9)	21 (32,3)	0,143
**Não HDL-c (mg/dL)** ^**†**^	95 (81-112)	92 (74-105,5)	0,063
**Não HDL-c indesejável**	81 (18,3)	8 (12,3)	0,234
**Glicose (mg/dL)**	85,9 ± 10,1	87,3 ± 7,7	0,302
**Triglicérides (mg/dL)** ^**†**^	66 (50-87,5)	62 (49,5-81)	0,444
**Triglicérides indesejável**	99 (22,4)	10 (15,4)	0,196
**Comportamentos e hábitos alimentares**			
Consumo regular^**#**^ de alimentos em frente à televisão	186 (45,8)	38 (62,3)	0,016*
Consumo regular^**#**^ de frutas	154 (36,2)	15 (23,4)	0,046*
Consumo diário de pelo menos 1 ultraprocessado	158 (37,4)	37 (59,7)	0,001*

As variáveis foram expressas em n (%) e média ± desvio padrão ou mediana (intervalo interquartil).^*†*^ Variáveis com distribuição não paramétricas. Comportamento sedentário foi definido como tempo sentando ≥ 3 horas. ^**#**^ Consumo regular de alimentos e frutas foi considerado para frequência ≥ 5 dias/semana. *Diferença estatisticamente significante. HDL-c: lipoproteína de alta densidade; IMC: índice de massa corporal; PAD: pressão arterial diastólica; PAS: pressão arterial sistólica.

*Fonte: Autores.*

A Tabela 3 apresenta a associação entre o comportamento sedentário e fatores de risco cardiometabólicos/hábitos alimentares na amostra de crianças e adolescentes fisicamente ativos. Após ajuste por variáveis potencialmente confundidoras, as crianças e adolescentes que apresentaram comportamento sedentário não demonstraram maior *odds ratio* para sobrepeso/obesidade e dislipidemia (TG, HDL-c e não HDL-c indesejáveis) quando comparadas aos que não apresentaram comportamento sedentário. Entretanto, as crianças e adolescentes que apresentaram comportamento sedentário tinham maior *odds ratio* para consumo regular de alimentos em frente à televisão e consumo de pelo menos um alimento ultraprocessado por dia. Além disso, também apresentaram menor *odds ratio* para consumo regular de frutas. A figura central sumariza os principais achados do manuscrito.

**Tabela 3:** – Associação entre comportamento sedentário e fatores de risco cardiometabólicos/hábitos alimentares em crianças e adolescentes fisicamente ativos

**Variáveis**	**OR (IC 95%)**	**p-valor**
**Sobrepeso/obesidade**		
Tempo sentado < 3 horas/dia	1	
Tempo sentado ≥ 3 horas/dia	0,72 (0,325-1,389)	0,283
**Triglicérides indesejável**		
Tempo sentado < 3 horas/dia	1	
Tempo sentado ≥ 3 horas/dia	0,63 (0,306-1,297)	0,210
**HDL-c indesejável**		
Tempo sentado < 3 horas/dia	1	
Tempo sentado ≥ 3 horas/dia	0,57 (0,323-1,019)	0,058
**Não HDL-c indesejável**		
Tempo sentado < 3 horas/dia	1	
Tempo sentado ≥ 3 horas/dia	0,63 (0,283-1,389)	0,250
**Consumo regular de alimentos em frente à televisão**		
Tempo sentado < 3 horas/dia	1	
Tempo sentado ≥ 3 horas/dia	1,96 (1,114-3,456)	0,020*
**Consumo regular de frutas**		
Tempo sentado < 3 horas/dia	1	
Tempo sentado ≥ 3 horas/dia	0,52 (0,278-0,967)	0,039*
**Consumo diário de pelo menos 1 ultraprocessado**		
Tempo sentado < 3 horas/dia	1	
Tempo sentado ≥ 3 horas/dia	2,42 (1,381-4,241)	0,002*

Modelo de regressão logística ajustada para sexo, raça/cor e estágio puberal. HDL-c, lipoproteína de alta densidade; IC 95%, intervalo de confiança de 95%; OR, odds ratio. Consumo regular de alimentos e frutas foi considerado para frequência ≥ 5 dias/semana. Comportamento sedentário foi definido como tempo sentado ≥ 3 h/dia. *Diferença estatisticamente significante.

*Fonte: Autores.*

**Figura Central:**
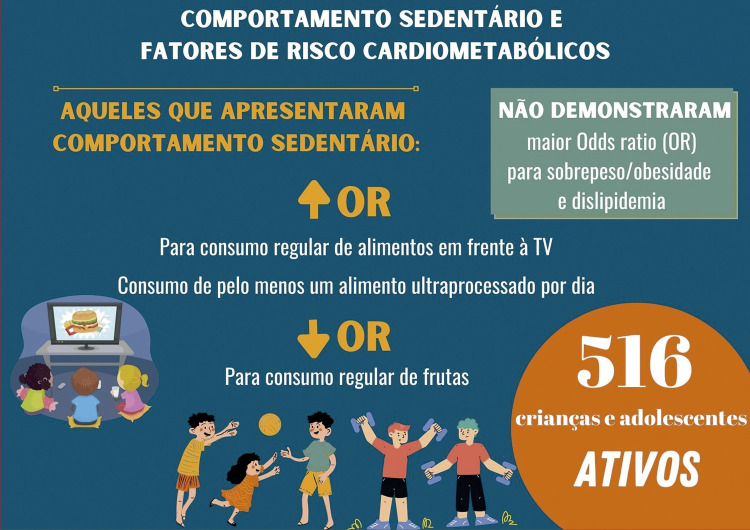
: Comportamento Sedentário, Hábitos Alimentares e Risco Cardiometabólico em Crianças e Adolescentes Fisicamente Ativos Principais achados do manuscrito.

Fonte: Autores.

## Discussão

Nosso estudo demonstrou que a adoção de comportamento sedentário não foi associada a fatores de risco cardiometabólicos em crianças e adolescentes fisicamente ativos. Contudo, a presença de comportamento sedentário foi associada à maior chance de se consumir regularmente alimentos em frente à televisão e de consumir pelo menos um alimento ultraprocessado por dia e à menor chance de se consumir frutas regularmente.

Nos últimos anos têm crescido o número de estudos avaliando a associação entre comportamento sedentário e fatores de risco cardiometabólicos.^24 , 25^ Um dos fatores que limita a comparação entre os estudos são as diferentes formas de classificação do comportamento sedentário, que pode ser por meio de medidas diretas como acelerômetros^24^ ou por meio de ferramentas indiretas (questionários), usando tempo de tela ou tempo sentado.^25^ Parte dos estudos classificam a adoção de comportamento sedentário quando o indivíduo fica, pelo menos, 2 h/dia sentado assistindo televisão, manuseando celular ou executando tarefas no computador.^26 - 28^ Nosso estudo foi realizado com crianças e adolescentes fisicamente ativos e, para aumentar a sensibilidade do instrumento de avaliação, optamos por utilizar como ponto de corte ≥ 3 h/dia de tempo sentado para determinação de comportamento sedentário. Esse mesmo ponto de corte também foi adotado por outros estudos na literatura.^23 , 25^

Recentemente, diversos estudos têm mostrado uma associação positiva entre adoção de comportamento sedentário e alterações de parâmetros cardiometabólicos em crianças e adolescentes.^24 , 25 , 29^ Santos et al.,^25^ estudando 457 adolescentes matriculados em escolas públicas no município de Curitiba-PR, demonstraram que os adolescentes que permaneceram maior tempo sentado apresentaram maiores valores de glicose, triglicerídeos e escore de risco cardiometabólico. Contudo, resultados de uma recente revisão sistemática e metanálise^29^ envolvendo estudos prospectivos com medida direta do comportamento sedentário (acelerometria), mostraram que não há associação entre comportamento sedentário e saúde cardiometabólica. De modo semelhante, o presente estudo também não evidenciou associação entre comportamento sedentário e fenótipos de composição corporal, hemodinâmicos e lipídicos, em crianças e adolescentes fisicamente ativos. A partir desses e de outros achados,^24^ é possível sugerir que os efeitos deletérios provocados pelo elevado tempo de comportamento sedentário possam ser minimizados pela prática regular de atividade física com intensidade moderada a vigorosa.

Além de alterações em parâmetros cardiometabólicos, alguns estudos têm avaliado a associação entre comportamento sedentário e distúrbios cardiometabólicos como obesidade,^30^ hipertensão^31^ e síndrome metabólica.^32^ Vicente-Rodríguez et al.,^30^ estudando 1.960 adolescentes espanhóis, demonstraram que o acréscimo de 1 h/dia no tempo de tela elevou em 15% o risco de sobrepeso/obesidade. Estudo recente avaliando 1.992 adolescentes iranianos, demonstrou que aqueles que despendiam mais tempo em atividades sedentárias apresentaram maior chance de serem hipertensos.^31^ Uma metanálise envolvendo 8 estudos transversais do *International Children’s Accelerometry Database* , demonstrou que o aumento de 1 hora no tempo de comportamento sedentário aumentou em 28% as chances de os indivíduos apresentarem síndrome metabólica. Contudo, quando o modelo foi ajustado para o tempo de atividade física moderada/vigorosa, a mesma associação não foi encontrada.^32^ No presente estudo não houve associação entre comportamento sedentário e distúrbios cardiometabólicos como obesidade e dislipidemia. Hipertensão e diabetes não foram avaliadas devido ao baixo percentual (< 0,5%) de crianças e adolescentes acometidos por essas enfermidades (dados não apresentados em tabelas). Existem alguns fatores que poderiam explicar os diferentes achados relatados acima. Nosso estudo só avaliou crianças e adolescentes fisicamente ativos. Dois dos estudos acima não ajustaram os dados para o tempo gasto com atividades físicas,^30 , 31^ o que pode ser um importante viés, visto que a atividade física pode atenuar os impactos do comportamento sedentário na saúde cardiometabólica. Outro fator que dificulta a comparação dos resultados é a forma de classificação do comportamento sedentário. Nosso estudo utilizou uma medida indireta, enquanto outros estudos utilizaram medida direta.^32^

Evidências sugerem que alguns contextos alimentares, como o hábito de realizar refeições assistindo à televisão, associam-se a uma pior qualidade da dieta. Cartanyà-Hueso et al.,^33^ Onita et al.^15^ e Rocha et al.^34^ conduziram estudos transversais com amostras representativas de crianças e adolescentes da Espanha, Reino Unido e Brasil, respectivamente, e evidenciaram que o hábito de comer em frente à tela associou-se a piores hábitos alimentares. Resultados de revisão sistemática e metanálise^35^ de estudos realizados com crianças mostraram associação entre comer enquanto assiste à televisão e consumo de alimentos/bebidas com alto grau de processamento industrial, como pizza, doces, salgadinhos de pacote e refrigerantes e, além disso, menor consumo de frutas e vegetais. Já foi sugerido na literatura que o hábito de fazer as refeições em frente à tela acarreta um mecanismo de “alimentação inconsciente”, além da exposição ao *marketing* persuasivo dos alimentos ultraprocessados. Essas formulações industriais apresentam baixo valor nutricional, uma vez que são densos em calorias e contém quantidades excessivas de gordura, açúcar e sal e, além disso, aporte insuficiente de fibras alimentares, proteínas, micronutrientes e compostos bioativos.^36 , 37^ Dessa forma, a maior praticidade dos alimentos ultraprocessados permite o consumo em qualquer lugar, sem a necessidade de preparo, cozimento ou uso de pratos ou talheres, ao mesmo tempo em que a criança ou adolescente assiste à televisão ou utiliza computador/ *video game* . Apesar de não termos encontrado associação entre comportamento sedentário e fatores de risco cardiometabólicos, hábitos alimentares inadequados associados ao sedentarismo na infância podem desencadear o aparecimento de doenças cardiometabólicas no futuro.^38 , 39^

O presente estudo apresenta algumas limitações. Primeiro, trata-se de um estudo transversal, portanto, não podemos inferir relação de causa e efeito. Segundo, a classificação de comportamento sedentário foi realizada por um método indireto (aplicação de questionário). Terceiro, os questionários foram preenchidos pelos próprios participantes, havendo alguma possibilidade de viés de informação. Entretanto, as questões utilizadas neste questionário são consideradas de fácil entendimento, sendo pequena a possibilidade desse viés. Quarto, os resultados do presente estudo não podem ser extrapolados para população geral de crianças e adolescentes do Brasil.

## Conclusão

Nosso estudo não demonstrou associação entre comportamento sedentário e fatores de risco cardiometabólicos em crianças e adolescentes fisicamente ativos. Contudo, o comportamento sedentário foi associado a hábitos alimentares inadequados. A partir disso, é possível sugerir que a prática regular de atividade física pode atenuar os efeitos deletérios do comportamento sedentário nos parâmetros cardiometabólicos de crianças e adolescentes.
