# Supplementary material for: Comportamento Sedentário, Hábitos Alimentares e Risco Cardiometabólico em Crianças e Adolescentes Fisicamente Ativos
Source: Arq Bras Cardiol. 2023 Jan 24;120(2):e20220357. [Article in Portuguese] doi: 10.36660/abc.20220357 (PMC9882420; doi:10.36660/abc.20220357)
Supplement: Translation

# Translation: Sedentary Behavior, Dietary Habits, and Cardiometabolic Risk in Physically Active Children and Adolescents

**Authors:** Paula Adriana dos Santos de Fontes, Jordana Herzog Siqueira, Haysla Xavier Martins, Polyana Romano Oliosa, Divanei Zaniqueli, José Geraldo Mill, Rafael de Oliveira Alvim

**Affiliations:** 1 Brazil Universidade Federal do Amazonas, Faculdade de Educação Física e Fisioterapia, Manaus, AM – Brazil; 2 Brazil Universidade Federal do Espírito Santo, Programa de Pós-Graduação em Saúde Coletiva, Vitória, ES – Brazil; 3 Brazil Universidade Federal do Espírito Santo, Programa de Pós-Graduação em Ciências Fisiológicas, Vitória, ES – Brazil; 4 Brazil Universidade Federal do Amazonas, Departamento de Ciências Fisiológicas, Manaus, AM – Brazil

**Keywords:** Sedentary Behavior, Cardiometabolic Risk Factors, Child, Feeding Behavior

## Abstract

**Background:**

Sedentary behavior has been associated with several cardiometabolic risk factors during childhood. However, little is known about the impact of sedentary behavior on the health and eating habits of physically active children and adolescents.

**Objective:**

To evaluate the association between sedentary behavior and cardiometabolic risk factors and eating habits in physically active children and adolescents.

**Methods:**

This cross-sectional study was conducted, including 516 physically active children and adolescents (10 to 18 years old; both sexes) enrolled in the social project “Estação Conhecimento-Vale” were evaluated. Biochemical and lifestyle variables (questionnaire) were collected. Sedentary behavior was determined indirectly (questionnaire), by using sitting time ≥ 3 hours per day as a cutoff point. A p-value < 0.05 was considered statistically significant for all tests.

**Results:**

Sedentary behavior was not associated with overweight/obesity (odds ratio = 0.72 [95% confidence interval (CI): 0.325-1.389]), hypertriglyceridemia (odds ratio = 0.63 [95% CI: 0.306-1.297]), low HDL cholesterol (odds ratio = 0.57 [95% CI: 0.323-1.019]), or high non-HDL cholesterol (odds ratio = 0.63 [95% CI: 0.283-1.389]). However, children and adolescents with sedentary behavior were more likely to regularly consume food in front of the television (odds ratio = 1.96 [95% CI: 1.114-3.456]) and to consume at least one ultra-processed food per day (odds ratio = 2.42 [95% CI: 1.381-4.241]). In addition, they were less likely to consume fruit regularly (odds ratio = 0.52 [95% CI: 0.278-0.967]).

**Conclusion:**

There was no association between sedentary behavior and cardiometabolic risk factors in physically active children and adolescents. However, sedentary behavior was associated with inadequate eating habits. Thus, we may suggest that the regular engagement in physical activity may attenuate the deleterious effects of sedentary behavior on the cardiometabolic parameters of children and adolescents.

## Introduction

Cardiovascular diseases are the leading cause of mortality worldwide and one of the main causes of disability.^1^Studies have shown that the presence of obesity and dyslipidemia during childhood and adolescence is associated with an increased risk of cardiovascular outcomes in adulthood.^2^According to studies with a representative sample of Brazilian adolescents, the prevalence of obesity and hypercholesterolemia was 8.4% and 21%, respectively.^3 , 4^Because of these alarming numbers, it is important to promote public health policies aimed at improving the quality of food, promoting regular engagement in physical activity, and fighting sedentary lifestyle during childhood and adolescence.

Sedentary behavior was once considered synonymous of physical inactivity. However, it is currently defined as the time spent with low-energy activities in a sitting position (≤ 1.5 metabolic equivalents [MET]).^5^Currently, there is a great concern about sedentary behavior, as it encompasses many activities in which children and adolescents usually engage for long periods during the day, such as using cell phones, computers, tablets, video games, and watching television. On this basis, some organizations from the United States have recommended that children limit leisure time in front of the television, cell phones, and computers.^6 , 7^Results of a recent systematic review and meta-analysis showed an association between long time spent in sedentary behavior and increased body weight, decreased physical fitness, inadequate eating habits, increased blood pressure, increased prevalence of chronic diseases, and worsening of lipid profile and glycemic control.^8 - 10^However, the impact of sedentary behavior on the cardiometabolic profile of physically active children and adolescents is still a matter of debate.

Eating habits, built and consolidated in childhood and adolescence, tend to remain throughout life,^11 , 12^and they are associated with the occurrence of chronic diseases in adulthood.^13^In recent years, there has been a global increase in the consumption of ultra-processed foods and beverages, as well as a reduction in the consumption of *in natura* foods (fruits and vegetables) by the child population.^14^Children who usually eat in front of the screen are more likely to consume foods and drinks with poor nutritional quality, as they are convenient and hyperpalatable.^15^Therefore, the nutritional quality of meals seems to be influenced by eating behaviors, such as eating alone in front of the screen.^16^

Given that most studies have investigated the impact of sedentary behavior on physically inactive children and adolescents, the present study sought to evaluate the association between sedentary behavior and cardiometabolic risk factors and eating habits in physically active children and adolescents.

## Methods

### Study design and sample selection

This cross-sectional study was conducted in a sample recruited from a social project called “Estação Conhecimento” (Serra, Espírito Santo State, Brazil) comprising a partnership between the public sector, the Vale mining company, and the community. The project is directed to schoolchildren and adolescents living in the vicinity of “Estação”. The majority of the families living in this region belong to the lowest socioeconomic class. All participants were regularly enrolled in public schools of the municipality, and they attended the project for a half-day period when they were not in school to receive additional classes (apart from regular school classes) and to have the opportunity to participate in sport and cultural activities.

From February 2014 to April 2016, 856 children and adolescents (6 to 18 years) attended the Cardiovascular Investigation Clinic located at the Federal University of Espírito Santo for clinical and biochemical exams and collection of information related to lifestyle. Of the total, 264 participants were excluded because of missing data (children younger than 10 years old did not answer the questionnaire related to physical activity and eating habits), and 76 because they were not physically active. Thus, 516 children and adolescents aged 10 to 18 years of both sexes were eligible ( Figure 1 ). Informed written consent and assent were obtained from parents or guardians in accordance with the Center for Health Sciences Ethics Committee (register number: 30385014.8.0000.5060).

**Figure 1:** – Sample flowchart.

Source: Authors.

### Demographic characteristics and pubertal stage

Demographic and lifestyle data (nutritional patterns, physical activity, and use of medications) were obtained through a questionnaire during an interview. Classification of race/color was self-reported and interviewer-reported. For prepubescent participants, interviewer’s impression was superimposed over the self-reported when divergent information was obtained. For pubescent/postpubescent participants, self-reported prevailed over the interviewer’s impression. Little divergent information was obtained in all pubertal stages. The phenotypes associated with race/color, such as skin color, hair shape, and facial traces were taken into account to classify the individuals as White, Black, or mixed race ( *pardo* , in Brazilian Portuguese).

The Tanner Scale was adopted to define the phases of sexual development according to secondary sex characteristics. The categories (pre-pubertal, pubertal, and post-pubertal) took into account the criteria validated for boys and girls in the studies by Marshall and Tanner.^17 , 18^

### Anthropometric variables

The anthropometric measurements were weight (Toledo Scale, Brazil, with accuracy of 0.05 kg) in barefoot individuals using only undergarments and height obtained using a wall-mounted stadiometer (Seca Stadiometer; Seca GmBH & Co, Hamburg, Germany) with accuracy of 0.1 cm. Muscle mass and fat mass (both in kilograms) were measured by multifrequency bioelectrical impedance analysis (MF-BIA8, InBody 230, Bioespace, South Korea). Body mass index (BMI) was calculated as the ratio between weight and squared height (kg/m^2^). BMI for age and sex percentile was calculated according to the standards provided by the World Health Organization and converted to Z scores. Children and adolescents with a BMI Z-score ≥ +1 were positioned in the category overweight/obesity.^19^

### Biochemical Variables

Blood collection was obtained by venipuncture after overnight fasting (8 to 12 hours) and sent to a central laboratory (Laboratório Tommasi, Vitória, Espírito Santo, Brazil) to determine serum concentrations of total cholesterol, high-density lipoprotein cholesterol (HDL-c), triglycerides (TG), and glucose. LDL-c was calculated by the Friedewald equation for those with TG ≤ 400 mg/dL. Non-HDL-c was calculated by subtracting HDL-c from total cholesterol. Undesirable biochemical parameters were considered as follows: TG > 90 mg/dL; HDL-c < 45mg/dL; non-HDL cholesterol > 120 mg/L.^7 , 20^

### Blood pressure measurements

Blood pressure (BP) was measured in the left arm using an automatic validated oscillometric device (Omrom 705CP; Intellisense, Tokyo, Japan) after a resting period of 5 minutes in the sitting position. Three consecutive readings with a minimum interval of 1 minute between measurements were taken from each patient. In the case of variation of more than 5 mmHg between the second and third measurement, a fourth measurement was required. Systolic blood pressure (SBP) and diastolic blood pressure (DBP) were calculated as the arithmetic mean of 2 measurements with a difference lower than 5 mmHg. Individuals with SBP or DBP above the 95th percentile were classified as hypertensive.^21^

### Sedentary behavior and eating habits

Data referring to sedentary behavior and eating habits were obtained through the options from the questionnaire applied by the National School Health Survey.^22^All participants in the present study were physically active, as they were engaged in physical exercises during sports training (soccer, swimming, judo, and athletics) within the “Estação Conhecimento” project. All the participants performed at least 180 minutes weekly of moderate-vigorous physical exercise. Information about sedentary behavior was collected using the following question: “On a typical weekday, how much time do you spend sitting, watching television, using a computer, playing video games, talking to friends or doing other sitting activities? (Not including Saturday, Sunday, holidays, and time spent sitting at school)”. Answer options: 1) less than 1 hours/day; 2) 1 to 2 hours/day; 3) 3 to 4 hours/day; 4) 5 to 6 hours/day; 5) 7 to 8 hours/day; 6) more than 8 hours/day. All the participants who spent ≥ 3 hours per day on these activities were classified as having sedentary behavior.^23 , 24^

Three variables on eating behavior were studied: (1) eating while studying or watching television; (2) consumption of ultra-processed foods and beverages (candy, stuffed cookies, sweets, chocolates, soft drinks, packaged snacks, sausages, salami, ham, nuggets); and (3) consumption of fresh fruit. Daily consumption of at least 1 ultra-processed food/drink and eating regularly while studying or watching television (≥ 5 days a week) were considered markers of inadequate eating behavior. Regular consumption of fresh fruit (≥ 5 days a week) was considered a marker of adequate eating behavior.

### Statistical analysis

Categorical variables were compared between sexes using the chi-square test and were reported as absolute and relative frequencies, whereas continuous variables with parametric distribution were compared using unpaired Student’s t test and reported as mean ± standard deviation. The continuous variables with non-parametric distribution were compared using Mann-Whitney test and reported as median (interquartile range). The Kolmogorov-Smirnov test was used to assess normality of variables. Multivariate binary logistic regression analysis was used to test the association between sedentary behavior (≥ 3 hours/day sitting) and overweight/obesity, dyslipidemia (high levels of TG and non-HDL-c, and low levels of HDL-c), and eating behavior (food consumption in front of the television and consumption of fruits and ultra-processed foods). All analyses were adjusted for sex, race/color, and pubertal stage. Analyses were performed using SPSS software (version 22) (Chicago, IL, USA), with the significance level set at 5%.

## Results

A total of 516 children and adolescents were evaluated, of which 318 (61.6%) male. The general characteristics of the sample are shown in Table 1 . Muscle mass and SBP were higher in boys than in girls, whereas body fat percentage and non-HDL-c were lower in boys than in girls. Also, the proportion of girls who consumed at least one ultra-processed food per day was higher than the proportion of boys

**Table 1:** – General characteristics stratified by sex in physically active children and adolescents

**Variables**	**Girls n=198**	**Boys n=318**	**p value**
**Age (years old)**	12.7 ± 1.81	12.7 ± 1.9	0.971
**Race/color**			0.002*
White	42 (21.2)	52 (16.4)	
Black	87 (43.9)	192 (60.4)	
Mixed race	62 (31.3)	70 (22.0)	
Others	7 (3.5)	4 (1.3)	
**Pubertal Stage**			0.330
Prepubertal	14 (7.4)	35 (11.2)	
Pubertal	151 (79.5)	242 (77.6)	
Postpubertal	25 (13.2)	35 (11.2)	
**BMI (Kg/m^2^)**	19.8 ± 3.9	19.4 ± 3.6	0.261
**Body fat (%)**	25.5 ± 7.7	20.2 ± 9.5	<0.001*
**Lean mass (Kg)**	18.7 ± 4.6	20.6 ± 6.6	0.001*
**Overweight/Obesity (%)**	42 (21.2)	55 (17.3)	0.268
**SBP (mmHg)**	103.6 ± 8.1	106.8 ± 9.2	<0.001*
**DBP (mmHg)**	62.2 ± 6.3	62.1 ± 6.8	0.900
**Hypertension (%)**	5 (2.5)	11 (3.5)	0.552
**Glucose (mg/dL)**	85.0 ± 8.7	86.8 ± 10.6	0.066
**HDL-c (mg/dL)** ^**†**^	49 (42-55)	47 (41-54)	0.254
**Low HDL-c**	73 (37.4)	133 (42.6)	0.247
**Non-HDL-c (mg/dL)** ^**†**^	98 (84-115)	92 (79-110)	0.008*
**High non-HDL-c**	44 (22.6)	45 (14.4)	0.019*
**Triglycerides (mg/dL)** ^**†**^	67 (55-88)	63 (47-85)	0.060
**High Triglycerides**	42 (21.6)	67 (21.5)	0.963
**Sedentary behavior**			
Sitting time ≥3 hours/day	30 (15.2)	35 (11.1)	0.168
**Eating behavior/habits**			
Regular consumption^#^of food in front of the television	83 (45.4)	141 (49.6)	0.167
Regular consumption^#^@of fruits	73 (38.4)	96 (32.2)	0.187
Daily consumption of at least 1 ultra-processed	88 (47.1)	107 (35.9)	0.015*

Variables were expressed as n (%) and mean ± standard deviation or median (interquartile range). †Variables with non-parametric distribution. #Regular consumption of food and fruits was considered for frequencies ≥ 5 days/week. *Statistically significant difference. BMI: body mass index; DBP: diastolic blood pressure; HDL-c: high-density lipoprotein cholesterol; SBP: systolic blood pressure.

*Source: Authors.*

Table 2 shows the comparison between groups in hemodynamic, biochemical, body composition, pubertal stages, and sociodemographic data. A higher proportion of post-pubertal individuals was observed in the group presenting sedentary behavior. The consumption of at least one ultra-processed food per day and the consumption of food in front of the television were more frequent in the group with sedentary behavior. In this group a lower proportion of individuals who regularly consumed fresh fruit was also observed.

**Table 2:** – General characteristics stratified by sedentary behavior in physically active children and adolescents

**Variables**	**<3 hours n=451**	**≥3 hours n=65**	**p value**
**Sex**			0.168
Girls	168 (37.3)	30 (46.2)	
Boys	283 (62.7)	35 (53.8)	
**Race/color**			0.150
White	79 (17.5)	15 (23.1)	
Black	243 (53.8)	36 (55.4)	
Mixed race	121 (26.8)	11 (16.9)	
Others	8 (1.8)	3 (4.6)	
**Pubertal Stage**			0.002*
Prepubertal	48 (11.0)	1 (1.5)	
Pubertal	344 (78.7)	49 (75.4)	
Postpubertal	45 (10.3)	15 (23.1)	
**BMI (Kg/m^2^)**	19.5 ± 3.8	19.7 ± 2.9	0.639
**Body fat (%)**	22.3 ± 9.3	21.9 ± 9.4	0.703
**Lean mass (Kg)**	19.7 ± 5.2	21.3 ± 5.2	0.060
**Overweight/Obesity**	87 (19.3)	10 (15.4)	0.451
**SBP (mmHg)**	105.6 ± 9.1	105.4 ± 6.9	0.901
**DBP (mmHg)**	62.3 ± 6.8	61.1 ± 4.8	0.070
**HDL-c (mg/dL)** ^**†**^	47.5 (41-55)	49 (43-54.5)	0.250
**Low HDL-c**	185 (41.9)	21 (32.3)	0.143
**Non-HDL-c (mg/dL)** ^**†**^	95 (81-112)	92 (74-105.5)	0.063
**High non-HDL-c**	81 (18.3)	8 (12.3)	0.234
**Glucose (mg/dL)**	85.9 ± 10.1	87.3 ± 7.7	0.302
**Triglycerides (mg/dL)** ^**†**^	66 (50-87.5)	62 (49.5-81)	0.444
**High Triglycerides**	99 (22.4)	10 (15.4)	0.196
**Eating behavior/habits**			
Regular consumption^#^of food in front of the television	186 (45.8)	38 (62.3)	0.016*
Regular consumption^#^of fruits	154 (36.2)	15 (23.4)	0.046*
Daily consumption of at least 1 ultra-processed	158 (37.4)	37 (59.7)	0.001*

Variables were expressed as n (%) and mean ± standard deviation or median (interquartile range). †Variables with non-parametric distribution. Sedentary behavior was defined as sitting time ≥ 3 hours. #Regular consumption of food and fruits was considered for frequencies ≥ 5 days/week. *Statistically significant difference. BMI: body mass index; DBP: diastolic blood pressure; HDL-c: high-density lipoprotein cholesterol; SBP: systolic blood pressure.

*Source: Authors.*

Table 3 presents the association between sedentary behavior and cardiometabolic risk factors/eating habits in the sample. After adjusting for potentially confounding variables, children and adolescents who showed sedentary behavior did not show a higher odds ratio for overweight/obesity and dyslipidemia (high TG, HDL-c, and non-HDL-c) when compared to those who did not show sedentary behavior. However, children and adolescents who showed sedentary behavior had higher odds ratio for regular consumption of food in front of the television and consumption of at least one ultra-processed food per day. In addition, they also had a lower odds ratio for regular fresh fruit consumption. The central figure summarizes the main findings of the manuscript.

**Table 3:** – Association between sedentary behavior and cardiometabolic risk factors/eating habits in physically active children and adolescents

**Variables**	**OR (95% CI)**	**p value**
**Overweight/Obesity**		
Sitting time <3 hours/day	1	
Sitting time ≥3 hours/day	0.72 (0.325-1.389)	0.283
**High Triglycerides**		
Sitting time <3 hours/day	1	
Sitting time ≥3 hours/day	0.63 (0.306-1.297)	0.210
**Low HDL-c**		
Sitting time <3 hours/day	1	
Sitting time ≥3 hours/day	0.57 (0.323-1.019)	0.058
**High Non-HDL-c**		
Sitting time <3 hours/day	1	
Sitting time ≥3 hours/day	0.63 (0.283-1.389)	0.250
**Regular consumption of food in front of the television**		
Sitting time <3 hours/day	1	
Sitting time ≥3 hours/day	1.96 (1.114-3.456)	0.020*
**Regular consumption of fruits**		
Sitting time <3 hours/day	1	
Sitting time ≥3 hours/day	0.52 (0.278-0.967)	0.039*
**Daily consumption of at least 1 ultra-processed**		
Sitting time <3 hours/day	1	
Sitting time ≥3 hours/day	2.42 (1.381-4.241)	0.002*

Logistic regression model adjusted for sex, race/color, and pubertal stage. HDL-c, high-density lipoprotein cholesterol; OR: odds ratio; 95%CI: 95% confidence interval. Regular consumption of food and fruits was considered for frequencies ≥ 5 days/week. Sedentary behavior was defined as sitting time ≥ 3 hours. *Statistically significant difference.

*Source: Authors.*

**Central Illustration:** : Sedentary Behavior, Dietary Habits, and Cardiometabolic Risk in Physically Active Children and Adolescents Main findings of the manuscript.

Source: Authors.

## Discussion

Our study showed that sedentary behavior was not associated with cardiometabolic risk factors in physically active children and adolescents. However, the presence of sedentary behavior was associated with higher odds of regular food consumption in front of the television and daily consumption of at least one ultra-processed food, as well as lower odds of regular fruit consumption.

In recent years, the number of studies analyzing the association between sedentary behavior and cardiometabolic risk factors has increased.^24 , 25^One of the factors that limits the comparison between studies is the different ways of classifying sedentary behavior, which can be done through direct measures such as accelerometers^24^or indirect tools (questionnaires), by using screen time or sitting time as parameters.^25^Part of the studies consider classifying as sedentary behavior when the individual spends at least 2 hours/day sitting watching television, using a cell phone, or performing tasks on the computer.^26 - 28^Our study was carried out with physically active children and adolescents. To increase the sensitivity of the assessment instrument, we chose to use a cutoff point of ≥ 3 hours/day of sitting time to determine sedentary behavior. This same cut-off point has also been preferred by other authors.^23 , 25^

Recently, several studies have shown a positive association between the adoption of sedentary behavior and changes in cardiometabolic parameters in children and adolescents.^24 , 25 , 29^Santos et al.,^25^studying 457 adolescents enrolled in public schools in the city of Curitiba, Paraná, Brazil, showed that adolescents who remained seated longer had higher glucose, TG, and cardiometabolic risk scores. However, the results of a recent systematic review and meta-analysis^29^involving prospective studies with direct measurement of sedentary behavior (accelerometry), showed that no association between sedentary behavior and cardiometabolic health. Similarly, the present study also did not show an association between sedentary behavior and body composition, hemodynamic, and lipid phenotypes in physically active children and adolescents. Based on these and other findings,^24^it is possible to suggest that the deleterious effects caused by a long period of sedentary behavior can be minimized by regular engagement in moderate/vigorous physical activity.

In addition to changes in cardiometabolic parameters, some studies have assessed the association between sedentary behavior and cardiometabolic disorders such as obesity,^30^hypertension,^31^and metabolic syndrome.^32^Vicente-Rodríguez et al.,^30^studying 1960 Spanish adolescents, reported that the addition of 1 hour/day of screen time increased the risk of overweight/obesity by 15%. A recent study with 1992 Iranian adolescents showed that those who spent more time in sedentary activities were more likely to be hypertensive.^31^A meta-analysis involving 8 cross-sectional studies of the International Children’s Accelerometry Database indicated that increasing 1 hour in the time of sedentary behavior increased the odds of metabolic syndrome by 28%. However, when the model was adjusted for time spent with moderate/vigorous physical activity, no such association was detected.^32^In the present study, there was no association between sedentary behavior and cardiometabolic disorders such as obesity and dyslipidemia. Hypertension and diabetes were not tested because of the low proportion (< 0.5%) of children and adolescents affected by these diseases (data not shown in tables). There are some factors that could explain the different findings reported above. Our study only considered physically active children and adolescents. Two of the studies above did not adjust the data for time spent on physical activities,^30 , 31^which may be an important bias, as physical activity might attenuate the impact of sedentary behavior on cardiometabolic health. Another factor that makes it difficult to compare the results is the method of classifying sedentary behavior. Our study used an indirect measure, while other studies used a direct measure.^32^

Evidence suggests that some dietary contexts, such as the habit of having meals while watching television, are associated with poorer diet quality. Cartanyà-Hueso et al.,^33^Onita et al.,^15^and Rocha et al.^34^conducted cross-sectional studies with representative samples of children and adolescents from Spain, UK, and Brazil, respectively, and showed that the habit of eating in front of the screen was associated with worse eating habits. Results of a systematic review and meta-analysis^35^of studies carried out with children showed an association between eating while watching television and the consumption of foods/beverages with a high degree of industrial processing, such as pizza, sweets, packaged snacks, and soft drinks, in addition to lower consumption of fruits and vegetables. It has been suggested that the habit of having meals in front of the screen leads to a mechanism of “unconscious eating”, in addition to exposure to persuasive marketing of ultra-processed foods. These industrial formulations have low nutritional value, since they are dense in calories and contain excessive amounts of fat, sugar, and salt, in addition to insufficient intake of dietary fiber, proteins, micronutrients, and bioactive compounds.^36 , 37^In this way, the greater practicality of ultra-processed foods allows consumption anywhere, without the need to prepare, cook, or use dishes or cutlery, while the child or adolescent watches television or uses a computer or video game. Although we did not find an association between sedentary behavior and cardiometabolic risk factors, inadequate eating habits associated with a sedentary lifestyle in childhood may trigger the appearance of cardiometabolic diseases in the future.^38 , 39^

The present study has some limitations. First, this is a cross-sectional study; therefore, we cannot infer a cause-and-effect relationship. Second, the classification of sedentary behavior was performed by an indirect method (questionnaire application). Third, the questionnaires were completed by the participants themselves, with some possibility of information bias. However, the questions used in this questionnaire are considered easy to understand, with little possibility of this bias. Fourth, because the sample was non-probabilistic, the results of the present study cannot be extrapolated to the general population of children and adolescents in Brazil.

## Conclusion

Our study showed no association between sedentary behavior and cardiometabolic risk factors in physically active children and adolescents. However, sedentary behavior was associated with inadequate eating habits. These results suggest that the regular engagement in physical activity may attenuate the deleterious effects of sedentary behavior on the cardiometabolic pararameters of children and adolescents.
